# Purchasing behavior and use of digital sports offers by CrossFit® and weightlifting athletes during the first SARS-CoV-2 lockdown in Germany

**DOI:** 10.1186/s13102-022-00436-y

**Published:** 2022-03-23

**Authors:** Nicole Meier, Till Nägler, Robin Wald, Annette Schmidt

**Affiliations:** Institut Für Sportwissenschaft, Fakultät Für Humanwissenschaften, Universität der Bundeswehr München, Werner-Heisenberg-Weg 39, 85577 Neubiberg, Germany

**Keywords:** CrossFit® performance, Weightlifting, COVID-19, Lockdown, Physical activity, Public health

## Abstract

**Background:**

To combat the spread of SARS-CoV-2, CrossFit® training centers, and fitness studios were closed during the first lockdown in Germany from mid-March until June 2020, and as a result, CrossFit® (CFA) or weightlifting athletes (WLA) faced a major challenge for the first time. Therefore, this study aimed to investigate the impact of the first lockdown on the training behavior and to analyze the way the athletes dealt with the new situation. In detail, we focus on habits of purchase and examine the acceptance of digital sports offers between CFA and WLA in response to the restrictions of the nationwide lockdown.

**Methods:**

An online survey was used to characterize the purchasing behavior and use of digital sports offers of CFA and WLA. In total, 484 volunteers (192 women, 290 men, 2 diverse) responded to the online questionary, allowing us to identify changes in training behavior and differences between the sports disciplines.

**Results:**

Our data shows both CFA and WLA purchase new equipment for a home gym and the use of digital sports increased significantly across all age groups. A comparison during the lockdown even showed that within the CFA, one group (n = 142) reported losing 5 kg or more of body mass, while the value of the WLA remained constant. On the one hand, the results indicate that despite the restrictions during the lockdown, CFA were may able to enhance health aspects by improving their body composition. On the other hand, this study shows that the training habits of both groups of athletes have changed significantly with the use of digital sports offers.

**Conclusions:**

We suppose that the great openness and the expansion of online sports offers during the first lockdown may change the sports industry in the future.

## Key points

- This study characterizes the first time the purchasing behavior and use of online sports offers of CrossFit® (CFA) or weightlifting athletes (WLA) by an online survey due to the closure of CrossFit® training centers and fitness studios to combat the spread of SARS-CoV-2 in Germany from mid-March to June 2020.
- The analysis of the data of 484 participants provides three significant changes comparing the training behavior before and during the first lockdown, first both athletes CFA and WLA bought new equipment for a home gym, second, the usage of digital sport offers increased, and a large group of CFA (n = 142) documented a weight loss of 5 kg and more.
- In conclusion, our data shows despite the restrictions during the COVID-19 lockdown CFA were able to achieve positive effects by practicing the CrossFit® sport and participating in digital sports offers.

## Background

To prevent the spread of SARS-CoV-2, all CrossFit® training facilities, and fitness studios were closed during the first lockdown in Germany, resulting in considerable restrictions with so far unknown consequences in practicing CrossFit® and weightlifting. While opportunities for public physical activity have been limited and the focus on improving health through physical activity may have been overshadowed by the combat against the COVID-19 pandemic, we will present how CrossFit® athletes (CFA) and weightlifting athletes (WLA) handle the situation during the first lockdown in Germany [1].

Within a period of only a few months, the SARS-CoV-2 virus has managed to spread across the world. This virus can spread by close contact, which includes large droplet spray and inhalation of microscopic droplets. The typical transmission routes of novel coronavirus include direct transmission (cough, sneeze, droplet inhalation transmission) and contact transmission with oral, nasal, and eye mucous membranes [2]. The fast-spreading of SARS-CoV-2 is also caused by a transmission that starts already two days before symptoms occur or even during infection without symptoms [3]. Government-imposed social distancing has become one of the primary ways of reducing the speed of spreading in many countries in recent months. The closing of all non-essential businesses is a central factor within this strategy [4]. Such non-essential businesses also include fitness studios and CrossFit® training centers. In mid-March, the first lockdown due to the COVID-19 pandemic was declared in Germany's federal states with a slightly noticeable offset [5]. The closure of the sports facilities lasted until June. Weightlifting and CrossFit® athletes did not have the opportunity to train as usual for around three months. From June onwards, strict restrictions still applied, which did not allow a return to the regular training as before [6].

The high-intensity interval training (HIIT) concept CrossFit® focuses on constantly varied functional movements executed at a high intensity. The training includes exercises from the main elements of gymnastics (e.g., Pull-Ups, Push-Ups, and Burpees), weightlifting (Power lifts, e.g. Back Squats, Deadlifts, and Olympic lifts, e.g., Snatch, Clean and Jerk), and cardiovascular activities (e.g., running, rowing, and jumping) usually performed as “workout of the day” (WOD) [7]. CrossFit® training is usually offered in affiliated training centers, where the required and extensive equipment (e.g., dumbbells, barbells, kettlebells, boxes, and jump ropes) and exercise machines such as rowing machines, air bikes and pull up bars are available. Nevertheless, the variety of CrossFit® training content allows athletes to train with considerably less equipment, e.g., only with body weight exercises, running, and jumping [8]. In contrast, weightlifting training emphasizes the use of free weight equipment (e.g., dumbbells and barbells) or weight machines to provide resistance to the exercise movement [9, 10]. Therefore, a minimum equipment with weights is essential for WLA. In limited cases, CFA are able to train without equipment, however, a entire CrossFit® training requires a full range of exercise equipment. Furthermore, access to high-weight equipment is a known problem for WLA [11]. Thus, both CFA and WLA are significantly affected by the closure of the fitness facilities in the execution of their regular training.

In history CrossFit® developed as a new trend sport in a short period of time, digital sports offerings and the formation of a virtual community contributed significantly to the rapid growth and building of the subculture around the trend sport [12]. So, in general, the training concept CrossFit® has good requirements for providing digital sports offers to train virtual at home and, accordingly we suspect a great openness to digital sports offerings among CFA.

However, to date, the impact of the training facility closures for CFA and WFL is unknown. Related studies of changes in training behavior in other sports in Europe report, for example, that the lockdown during the COVID-19 pandemic led to reduced training behavior overall among Spanish basketball players or reduced training time on the ball by Austrian soccer players [13, 14]. We assume that the athletes of both sports have equipped themselves with equipment during the lockdown to train as usual at home. However, the absence of the CrossFit® community in the training facility might be a potential influencing factor to affect training behavior or participation in digital training offerings.

We, therefore, ask, how the nationwide lockdown in Germany from mid-March until June 2020 chances the training behavior of CFA and WLA. To provide a detailed insight into how the athletes dealt with the new situation and to identify the differences between the disciplines, we report a characterization of the purchasing behavior and use of digital sports offers by CFA and WLA during the first SARS-CoV-2 lockdown. In addition, based on our online survey, we present differences in training frequency and changes in body mass during this period.

## Methods

### Data collection procedure by online survey

To characterize the training behavior of CFA and WLA before and after the first lockdown in Germany, the study was conducted using a common online survey tool that met the university’s ethics and privacy policy. For investigation, we developed a questionnaire based on standardized scales and the current state of the literature and validated by fifteen sports scientists according to the method of Gravettter and Forzano [15]. Following validation and two months after the first lockdown was declared in Germany, the questionnaire was online available at www.soscisurvey.de for 16 days (18th of May till 2nd of June 2020), and the link was shared on local CrossFit® platforms, Weight training platforms, and social media.

### Measurements

The first item on the questionnaire included a choice question about which sport the participants performed, CrossFit®, weightlifting, or neither. Thus, the participants were selected on the criteria of performing any of both sports. After collecting common anthropometric and demographic data, the participants were asked about their training behavior and reason for sport in the previous period of the first lockdown and during the current lockdown period, which began in Germany on March 15, 2020. To analyze changes in purchasing behavior and use of digital sports offerings before and during the first lockdown, the survey includes items regarding equipment at home and attendance and motivation for digital sports.

### Statistical analysis

All data are presented as mean ± standard deviation (SD). For data interpretation, IBM SPSS version 26 (IBM, Armonk, NY, USA) was used. The normality was tested using the Shapiro–Wilk test and Q–Q plots. For the test of sampling adequacy, a Kaiser–Meyer–Olkin (KMO) analysis was performed. To compare the training behaviors, normally distributed variables were analyzed using the Students T-Test. For ordinal scaled or non-normal distributed variables, Mann–Withney-U tests were carried out. Nominal-scaled variables were analyzed using Chi-square. The level of statistical significance (α) was set at 0.05.

## Results

### Demographic and anthropometric data of the participants

To characterize the impact of the first lockdown on the training behavior, in total, 484 athletes (59.9% men, 39.7% women, and 0.4% diverse) practicing CrossFit® or weightlifting participated in this survey. The average age was 31 years (range 18–65 years), with comparable average ages between males (18–65 years) and females (19–63 years). Demographic data showed that most participants had higher education and more than half were employees, while 1/3 were students, with an average weekly working time of all participants of 37 h before the COVID-19 pandemic. A detailed overview of the descriptive athlete’s characteristics is given in Table 1.

**Table 1:** Overview about demographic and anthropometric data of the participants

	Total	Women	Men	Diverse
Completed questionnaires	100% (484)	39.7% (192)	59.9% (290)	0.4% (2)
Age (years)	31 (18–65)	33 (19–63)	29 (18–65)	32 (31–33)
Hight (cm)	176 (155–201)	168 (155–188)	181 (167–201)	162 (160–164)
Weight (kg)	76 (48–130)	65 (48–116)	84 (57–130)	69 (57–80)
Educational degree				
Secondary school	3.5% (17)	4.7% (9)	2.7% (8)	–
Completed vocational training	6.4% (31)	7.8% (15)	5.5% (16)	–
High school	28.9% (140)	18.8% (36)	35.9% (104)	–
Bachelor	25.0% (121)	23.4% (45)	26.2% (76)	–
Master	33.1% (160)	42.2% (81)	26.9% (78)	50% (1)
Doctor	3.1% (15)	3.1% (6)	2.8% (8)	50% (1)
Employment				
Student	33.0% (160)	21.3% (41)	41.0% (119)	–
Employee	53.1% (257)	62.5% (120)	46.6% (135)	100% (2)
Official	8.3% (40)	9.4% (18)	7.6% (22)	–
Self-employed	4.5% (22)	5.2% (10)	4.1% (12)	–
Homemaker	0.4% (2)	1.0% (2)	–	–
Pensioner	0.2% (1)	0.5% (1)	–	–
Unemployed	0.4% (2)	–	0.7% (2)	–
Income, net monthly (€)				
Less than 500	1.2% (6)	2.1% (4)	0.7% (2)	–
500–1500	9.7% (47)	11.5% (22)	8.6% (25)	–
1500–2500	37.4% (181)	34.9% (67)	39.0% (113)	50.0% (1)
2500–3500	30.0% (145)	27.6% (53)	31.7% (92)	–
3500–4500	8.3% (40)	8.3% (16)	8.3% (24)	–
4500 and more	4.8% (23)	3.6% (7)	5.2% (15)	50.0% (1)
Not specified	8.7% (42)	12.0% (23)	6.6% (19)	–
Regular working time per week (h)	37 (0–60)	39 (0–60)	37 (0–60)	40 (39–40)
Training experience				
Less than 3 months	3.1% (15)	0.5% (1)	4.8% (14)	–
3–6 months	5.8% (28)	4.4% (8)	6.9% (20)	–
6–12 months	9.5% (46)	11.5% (22)	8.3% (24)	–
12–24 months	17.1% (83)	21.9% (42)	14.1% (41)	–
More than 24 months	64.5% (312)	62.0% (119)	65.9% (191)	100% (2)
Training days per week				
1	3.9% (19)	3.1% (6)	4.5% (13)	–
2	14.9% (72)	16.1% (31)	14.1% (41)	–
3	29.8% (144)	31.8% (61)	27.9% (81)	50% (1)
4	26.4% (128)	26.0% (50)	26.9% (78)	–
5 and more	25% (121)	22.9% (44)	26.6% (77)	50% (1)

If units are given, first value shows the mean, and the range is given in brackets. For the rest the percentage is given, and the total number is given in brackets

While most participants trained three or more days per week for more than two years, of those 266 reported CrossFit® [hereafter referred to as CrossFit® athletes (CFA)] and 218 reported weightlifting [hereafter referred to as weightlifting athletes (WLA)] as their primary sport. Since the study aims to characterize the effects of the first lockdown by showing the differences in the behavior of CFA and WLA, we first present the comparison between the two groups in order to be able to show the different changes due to the restrictions.

### Comparison of athletes doing CrossFit® or weightlifting

Our data shows that women did more likely CrossFit® and men weightlifting (*p* < 0.001). Regardless, both groups had comparable training experiences, and most of them had an experience of more than two years (*p* = 0.055). Although CFA had significantly higher working time (*p* = 0.003) before the first lockdown, however, both groups had similar working times during the lockdown (*p* = 0.164). In comparison, CFA trained more days per week (*p* = 0.04) before the first lockdown as well as during the first lockdown (*p* = 0.005), see Table 2.

**Table 2:** Comparison of attendees doing CrossFit® or weightlifting as a primary sport

	CrossFit®	Weightlifting	*p* value
Gender			< 0.001
Women	68.2% (131)	31.8% (61)	
Men	45.9% (133)	54.1% (157)	
Diverse	100% (2)	0% (0)	
Training experience			0.055
Less than 3 months	2.6% (7)	3.7% (8)	
3–6 months	4.9% (13)	6.9% (15)	
6–12 months	12.0% (32)	6.4% (14)	
12–24 months	19.9% (53)	13.8% (30)	
More than 24 months	60.5% (161)	69.3% (151)	
Working time per week (before)			0.003
Less than 10 h	1.6% (4)	1.9% (4)	
10–19 h	2.0% (5)	6.1% (13)	
20–29 h	10.6% (27)	9.9% (21)	
30–39 h	26.8% (68)	39.0% (83)	
40 h and more	59.1% (150)	43.2% (92)	
Working time per week (during)			0.164
Less than 10 h	8.6% (22)	3.8% (8)	
10–19 h	8.2% (21)	9.9% (21)	
20–29 h	12.1% (31)	10.8% (23)	
30–39 h	27.0% (69)	33.5% (71)	
40 h and more	44.1% (113)	42.0% (89)	
Training days per week (before)			0.04
1	2.6% (7)	5.5% (12)	
2	14.7% (39)	15.1% (33)	
3	27.4% (73)	32.6% (71)	
4	25.2% (67)	28.0% (61)	
5 and more	30.1% (80)	18.8% (41)	
Training days per week (during)			0.005
1	9.1% (21)	9.1% (17)	
2	15.5% (36)	20.4% (38)	
3	25.4% (59)	29.0% (54)	
4	18.5% (43)	25.8% (48)	
5 and more	31.5% (73)	15.6% (29)	
Additional sports (before)			0.004
None	53.0% (141)	42.2% (92)	
Endurance	25.2% (67)	27.5% (60)	
Ballgames	7.5% (20)	12.8% (28)	
Climbing	0.8% (2)	3.2% (7)	
HIIT, other	0.8% (2)	0.9% (2)	
Fight sport	2.6% (7)	6.4% (14)	
Dancing	2.3% (6)	2.8% (6)	
Weightlifting	5.3% (14)	0.5% (1)	
Yoga	1.5% (4)	2.8% (6)	
Others	1.1% (3)	0.9% (2)	
Additional sports (during)			0.003
None	49.6% (132)	39.0% (85)	
Endurance	28.9% (77)	28.9% (63)	
Ballgames	6.0% (16)	12.4% (27)	
Climbing	0.8% (2)	3.2% (7)	
HIIT, other	0.8% (2)	0.5% (1)	
Fight sport	2.6% (7)	5.5% (12)	
Dancing	1.9% (5)	2.8% (6)	
Weightlifting	4.9% (13)	0.5% (1)	
Yoga	2.6% (7)	3.2% (7)	
Home workouts	1.1% (3)	3.2% (7)	
Others	0.8% (2)	0.9% (2)	

*p* values were calculated using Chi-squared test (before: time before lockdown; during: time during lockdown). If units are given, first value shows the mean, and the range is given in brackets. For the rest the percentage is given, and the total number is given in brackets

In Addition, athletes were asked about additional sports before and during the lockdown. The results indicate that 53% of the CFA and 42.2% of the WLA did not do any additional sport besides their main sport before the lockdown. Whereas 25.2% of the CFA and 27.5% of the WLA performed endurance training additionally.

### Changes during the first lockdown

**Fig. 1:**
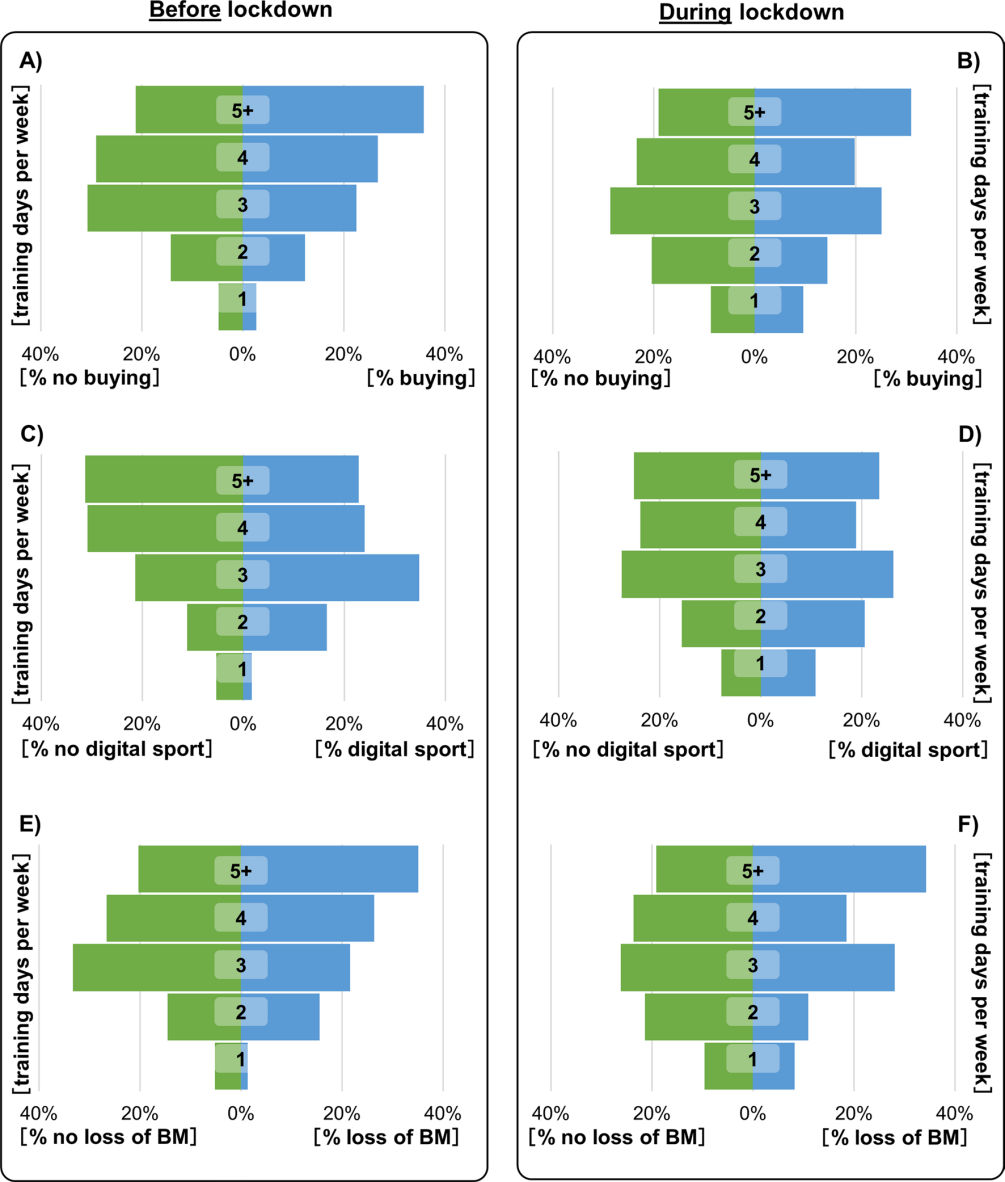
The training days (1–5 +) of all athletes before (left box) and during (right box) the first lockdown in Germany were analyzed in combination with buying of new equipment (**A**, **B**), usage of digital sport offers (**C**, **D**) and the loss of 5 kg body mass (BM) or more (**E**, **F**)

Three significant changes were observed compared before and during the first lockdown. Athletes bought new equipment for a home gym, the usage of digital sport offers increased, and a large proportion documented a weight loss of 5 kg and more. All these three observations were associated with their weekly training frequency. Most athletes who bought new equipment for a home gym (36%) trained 5 days or more per week before the lockdown (Fig. 1A) as well as during the lockdown (31%) (Fig. 1B).

The training frequency of the athletes who did not buy any equipment did not change significantly before the lockdown vs during the lockdown; this group (31% and 29%) continued to train mainly 3 days per week (Fig. 1A, B). The number of athletes (27%) who trained 4 days a week before the lockdown and bought equipment for a home gym decreased during the lockdown (20%) (Fig. 1A, B). Most athletes who used digital sports offers trained 3 days a week before the lockdown (35%) versus 26% during the lockdown. Those who trained 5 days and more a week before the lockdown (23%) and used digital sports offers also trained 5 days or more per week during the lockdown (23%) (Fig. 1C, D). Most athletes who lost 5 or more kg of body mass during the lockdown trained 5 days or more per week before (35%) as well as during the lockdown (34%) (Fig. 1E, F).

The practice of additional sports also shifted during the lockdown in a manner that the percentages of athletes not practicing additional sports decreased to 49.6% of CFA and 39.0% of WLA, and the percentages of athletes practicing endurance training increased analogously to 28.9% and 28.9%, respectively.

### General aspects of the two groups of athletes with and without purchased equipment

Athletes were asked if they bought additional training equipment during the lockdown. Those, who bought equipment were more frequently in short-time work (10.2% vs. 3.9%, *p* = 0.011) and more likely CFA (49% of all CFA and 39% of all WLA, *p* = 0.043). Before lockdown athletes who bought new equipment did more often sports (main group of 35.8% did 5 sessions or more per week, whereas the others did with 30.7% mainly 3 times training, *p* = 0.016), but training frequency during lockdown did not show significant differences (*p* = 0.55). In addition, a larger proportion in this group went usually to a gym (88.8% vs. 76.2%, *p* = 0.001), did less endurance training before the lockdown (40.6% vs. 51.9%, *p* = 0.021), less body weight training (33.2% vs. 50.2%, *p* ≤ 0.001), and did have had own equipment at home before (27.8% vs. 37.7%, *p* = 0.033). Usage of digital sport offers were less often stated (11.8% vs. 19.0%, *p* = 0.028), but more often training with a partner (46.5% vs. 36.4%, *p* = 0.023).

### Digital sports offers depending of training days, age, and nutrition

**Fig. 2:**
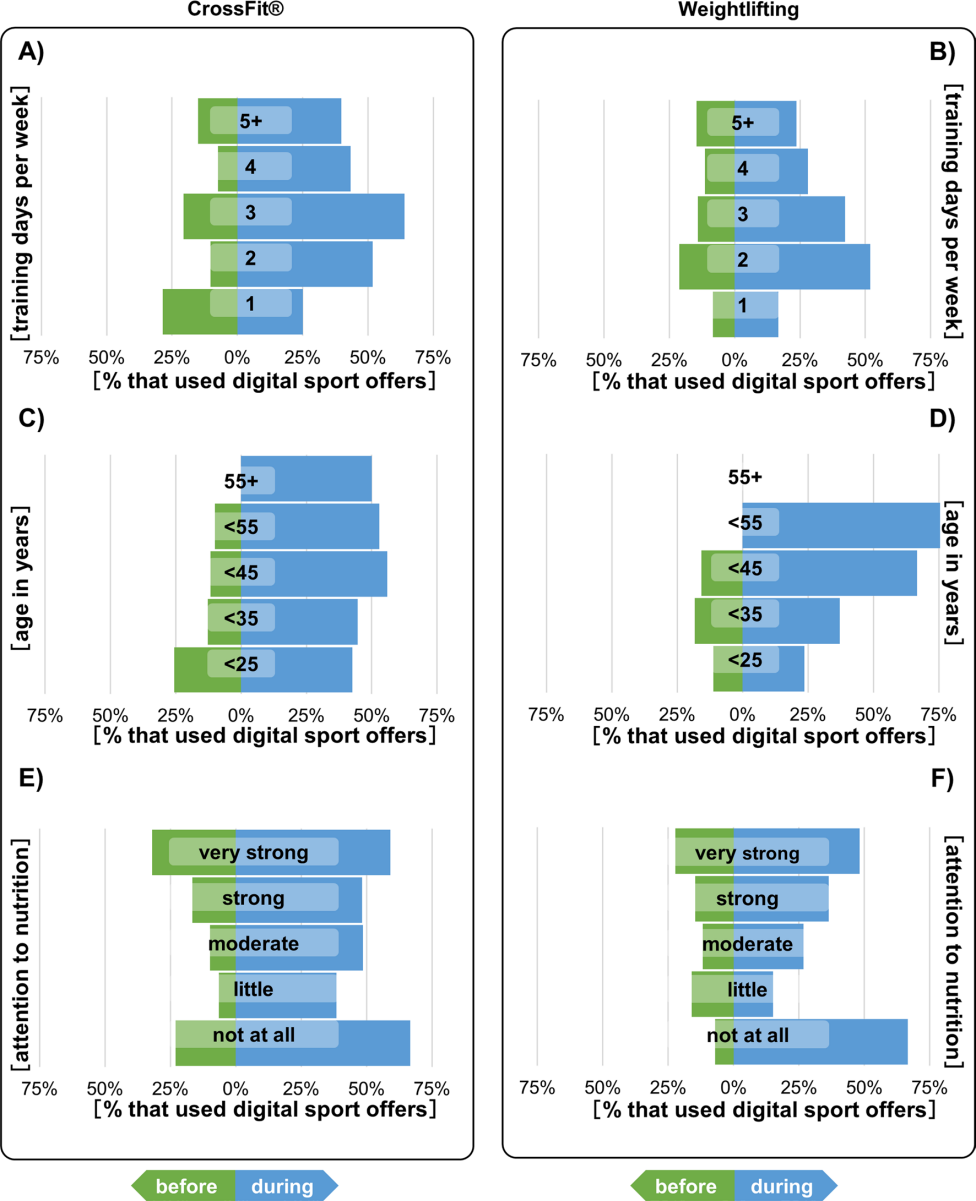
Usage of digital sport offers before (green bars) and during (blue bars) the first lockdown in Germany (indicated as ‘before’ and ‘during’). Participants were subdivided according to their primary sport (CrossFit® or weightlifting). The groups were separated on the amount of training days before the lockdown (**A**, **B**) the age (**C**, **D**), and how much they pay attention to their nutrition (**E**, **F**)

In the following part, we focused on the use of digital sports offers depending on the number of training days per week, age, and attention to nutrition. For all athletes (CFA and WLA) an increase in using digital sports offers was observable during the lockdown, independent from their training days (except CFA who trained 1 day per week). That increase was higher for CFA. The highest increase was observed for athletes, that trained 2 or 3 days per week. Of the CFA who trained 5 days a week, 15% already took advantage of digital sport offers before the lockdown, while it was 40% during the lockdown. Of those WLA who trained 5 days a week, also 15% took advantage of digital sport offers before the lockdown and 24% during lockdown (Fig. 2A, B).

If one looks at the use of digital sports offers in relation to age, it is noticeable that athletes over 55 years of age did not take advantage of digital offers before the lockdown (0%). Among the CFA > 55, usage increased to 50% during the lockdown and remained at 0% for the WLA. Among the under 55-year-olds, 10% of the CFA and 0% of the WLA took advantage of digital sports offers before the lockdown. During the lockdown, usage increased to 53% for the CFA and 100% for the WLA. Among the CFA < 45, 12% used digital sport offers before the lockdown and 56% during the lockdown, among the WLA < 45 were 16% before and 67% during the lockdown. Among the < 35-year-olds, 13% of the CFA and 18% of the WLA used digital sports offers before the lockdown. During the lockdown, it was 45% of the CFA and 37% of the WLA. In the age group < 25 were 26% of the CFA and 11% of the WLA before the lockdown and 43% of the CFA and 24% of the WLA during the lockdown (Fig. 2C, D). When looking at the use of digital sports offers depending on diet, there was also an increase across all categories. Those CFA who said that they pay attention to their diet “very strong”, 32% took part in digital sport offers before and 59% during the lockdown. Of WLA in the same category, 22% took part in digital sports offers before and 48% during the lockdown. The greatest increase was among those CFA and WLA who said they pay attention “not at all” to their diet. Here it was 67% of the CFA and WLA who took part in digital sports offers during the lockdown (Fig. 2E, F).

### Losing 5 kg and more body mass

**Fig. 3:**
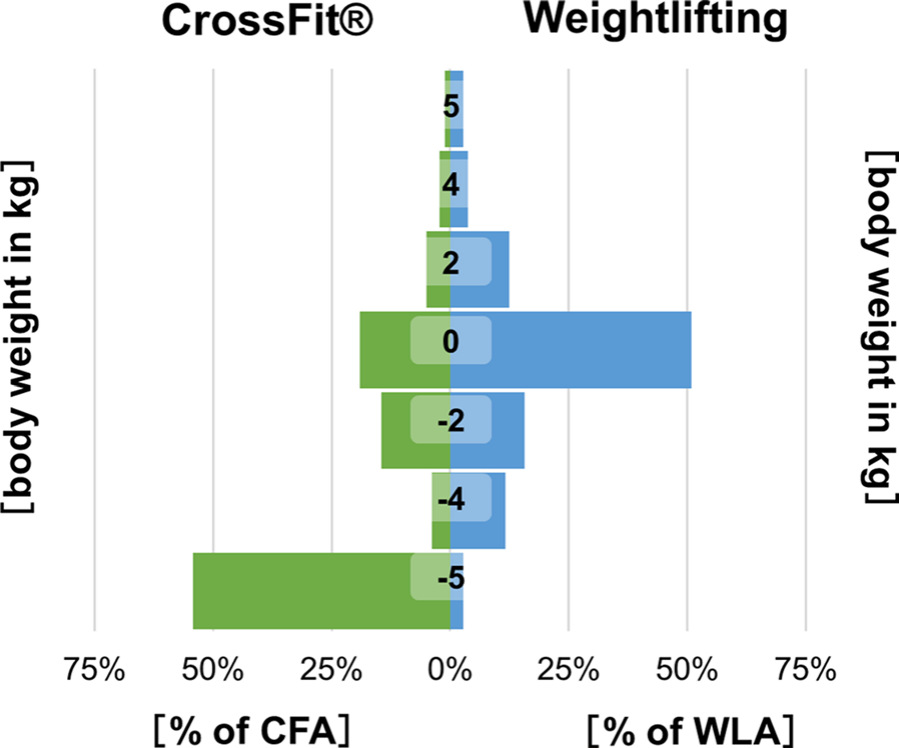
Distribution of weight changes during the lockdown divided into athletes doing CrossFit® (green bars) or weightlifting (blue bars). Meaning of the numbers within the bars: 5: weight increase by 5 kg and more; 4: weight increase by around 4 kg; 2: weight increase by around 2 kg; 0: 0 kg weight more or less = unchanged; − 2: weight decrease by around 2 kg; − 4: weight decrease by around 4 kg; − 5: weight decrease by 5 kg or more)

For the majority of the athletes (70% divides into 51% of WLA and 19% of CFA), no change in body mass was recorded. However, what stands out was a group of 142 respondents (29.7%) who practice CrossFit® and who answered they had lost more than 5 kg body mass, see Fig. 3.

This group seemed to be different from the rest (hereafter referred to as CF5). The athletes in the CF5 group were often women (53.3%), which explains why this group is significantly smaller and lighter. The CF5 group were older (33.9 vs 29.0 years, *p* < 0.001) and trained more likely to improve health (81.0% vs 72.0%, *p* = 0.039) and less to build muscles or aesthetics. In CF5, the mean income was higher, but the proportion of people in short-time work (12.7% vs 3.9%, *p* < 0.001) or compulsory leave (7.0% vs 1.8%, *p* = 0.004) because of the lockdown was increased, see Table 3.

**Table 3:** Overview about all significant differences of CFA who declared to have lost 5 kg or more (CF5 group) with all the others

	CF5 group	All others	*p* value
Age (years)	33.9 ± 8.4	29.0 ± 7.8	< 0.001
Gender			< 0.001
Women	53.30%	33.60%	
Men	46.50%	65.80%	
Diverse	0%	0.60%	
Height (cm)	173 ± 8.8	177 ± 8.9	< 0.001
Weight (kg)	72.8 ± 12.5	78.0 ± 13.6	< 0.001
Exercises with a partner	54.90%	35.70%	< 0.001
Exercises to build muscle	69.70%	78.60%	0.039
Exercises to improve aesthetics	38.00%	53.30%	0.002
Training-days per week			
Before	3.8 ± 1.1	3.4 ± 1.1	0.004
During	3.6 ± 1.2	3.2 ± 1.4	0.005
Trains to improve health			
Before	81.00%	72.00%	0.039
During	87.90%	78.30%	0.018
Short time work because of the lockdown	12.70%	3.90%	< 0.001
Compulsory leave because of the lockdown	7.00%	1.80%	0.004
Income, net monthly (€)			0.001
Less than 500	2.40%	1.00%	
500–1500	6.50%	11.50%	
1500–2500	31.70%	45.40%	
2500–3500	40.70%	29.70%	
3500–4500	8.10%	9.30%	
4500 and more	10.60%	3.20%	

Before meaning information on the situation before the lockdown; during meaning information on the situation during the lockdown. Values are given as a percentage or mean value with standard deviation

## Discussion

In this study, we characterized for the first time in detail the changes in training behavior of CFA and WLA during the first lockdown from mid-March until June 2020 of the COVID-19 pandemic in Germany. We found three significant changes comparing the training behavior before and during the first lockdown. First, both CFA and WLA bought new equipment for a home gym, second, the usage of digital sports offers increased, and a large group of CFA documented a weight loss of 5 kg and more. The first lockdown beginning in mid-March 2020 in Germany was the first time that training centers were closed nationwide and, due to the short history of CrossFit®, it was also the first time that athletes could not train as usual in their training centers. So, our study describes for the first time the impact of the first lockdown on CFA and WLA and analyses the differences of the training concepts in this context.

We focused in this study on CFA and WLA as both need a lot of equipment. Garage gyms are very rare in Germany, and people most often perform these sports in sports facilities, where the necessary equipment is available [11]. So, both groups were hard hit by the restrictions to combat the spread of SARS-CoV-2. Therefore, we were interested in how the two groups handled the situation, equally or differently, and what factors might impact possible differences. While weightlifting is usually performed alone or with a partner, CrossFit® is a group sport characterized by strong social interaction and a sense of community [11, 16, 17].

Thus, by comparing both disciplines, our results show that CFA and WLA differ in many ways. As weightlifting has less variation than CrossFit®, those athletes perform additional sports more often, like Endurance and ballgames. CFA are more common women, have longer working hours per week, and train more often per week. CFA's high training volume per week is consistent with previous studies describing over 6 training hours per week on average for German and American athletes [18]. Our survey indicates CFA train more days per week in comparison with WLA, probably caused by shorter workouts or training time per session. In addition to the closing of all non-essential businesses during the first lockdown, many employees used the opportunity to work from home to reduce further personal contacts [19]. We assume that shorter workouts like ultra-short CrossFit® workouts shown by Meier et al. are better integrable into breaks of home office work [20].

Overall, 49% of CFA and 39% of WLA purchase new equipment during lockdown to train at home, in line with our expectations, as both sports require a large amount of equipment. Due to the large and unexpectedly high demand for sports equipment, it led to up to 90% sold out online and in stores [21]. Due to this fact, our results may show a bias, as not every athlete had the opportunity to purchase new equipment.

An unmistakable trend during the first lockdown was the increasing availability of digital sports content and so, across all age groups, were we able to observe a significant increase in the usage of digital sports offers. To continue offering a variety of exercise and training activities, several digital training tools have been developed to date. Generally, 3 types of offers can be distinguished: live streaming of digital training courses, digital distribution of written training units, and the production of videos that can be viewed by members independent of time [22].

Scientifically, the status of such services is currently unclear. In a systematic meta-analysis, Romeo et al. concluded that digital interventions by smartphone apps have only a nonsignificant, positive influence on measured physical activity [23]. The same is reported in a meta-analysis focused on older persons [24], and in comparison, of several concepts for young adults [25]. This lack of positive physical impact may be because such approaches do not work or are not mature enough. Nevertheless, in 2020 a lot of new digital concepts have come up [26]. Many CrossFit® training facilities were forced to move their service online and as a result, they launched digital training provided to their members. There were also occasional attempts to achieve interactions and connections in the respective groups via virtual platforms and social media [27].

As this is a new and fast-evolving phenomenon, there is as yet no scientific evidence of the value of such services and the benefits that athletes receive. Nevertheless, we observed strong participation of CFA in digital sport offerings, especially among older athletes (> 55 years) who may not have previously experienced these. In contrast, WLA in this age group did not participate in any online sports offers. To explain this result, we suggest that, based on the assumption weightlifting workouts are easier to program than CrossFit® workouts [28], WLA already know how to train themselves without participating in digital sports.

The larger acceptance of digital sport offers reflects a higher sense of community among CFA and strong social interaction, in accordance with previous studies [16, 29]. A related conclusion was reached in the study by Redwood-Brown et al. so far. They reported that athletes who were already practicing CrossFit® before had not altered their training behavior during the lockdown, a fact they attributed to the increased adherence associated with CrossFit® [30]. This is consistent with further findings suggesting that one of the most important interventions for a CrossFit® training facility should be, especially during the COVID-19 pandemic, to establish a Facebook and Instagram community for its members. These online communities have been shown to provide great value to the athletes both before and during the lockdown, such as social and motivational benefits [27, 31].

Another factor that may explain the increased use of digital services of CFA is that a variety of gymnastics and cardiovascular exercises can be adapted to train at home [32], while WLA relies heavily on free weights or weight machines, which were only partially available at home. Thus, we hypothesize that, in addition to the sense of community, the modality of online training and the practicability at home influence on participation, although based on our data, where we did not determine the specific requirements of the digital training athletes participate in, we are unable to answer this question.

The most surprising result of our study was that one group of CFA (n = 142) achieved a weight loss of 5 kg or more. Interestingly, the majority of this group were women and trained CrossFit® to improve their health. For this reason, we assume, that the group of CF5 may improve their body composition in contrast to the general population, which is characterized by increased physical inactivity during the lockdown, resulting in weight gain and other negative health effects [33, 34]. To consider probable explanations for the weight loss results of group CF5, other influencing factors may need to be included. So, a study regarding behavior change during COVID-19 pandemics found that the group that was more active during the lockdown also changed their dietary habits toward a healthier profile [35]. As our data also show that group CF5 spent more time at home due to increased short-time work or compulsory leave as a result of the lockdown, we suspect that more time and focus on a healthier lifestyle as well as increased CF training time may have resulted in this outcome. Nevertheless, due to the restrictions of the first lockdown were are unable to verify the weight changes of CFA by measurements, which affects the conclusion of our study. However, despite this, both types of athletes usually track their body mass very detailed, so our data provide a helpful assessment of how the restrictions of the first lockdown impacted a number of the CFAs we studied.

The trend towards training at home experienced a massive increase during the lockdown, and we were also able to observe this during this survey. For this reason, the study is not without limitations, despite the novel findings. What we had not considered while designing the study was the extraordinary situation that many sports equipment retailers had sold out their everyday items for months. Thus, has undoubtedly had an impact on the number of purchases.

Overall, our results indicate potential benefits in CrossFit® and weightlifting sport during the fist lockdown, so we suggested that practicing CrossFit® may improve body composition despite the restriction to combat the spread of SARS-CoV-2. In general, we emphasize here the positive health aspects of practicing CrossFit® or weightlifting as opposed to overall observations regarding the physical activity of adults during lockdown [36]. In addition, increased digital sports offerings allow training facilities to reach more potential customers [37], and athletes have the opportunity to perform CrossFit® regardless of where they are located. In Particular, in CrossFit® sport, our results show a great acceptance of digital sport offers, across all ages groups. However, evidence of the positive physical effects and performance enhancement of digital sports is still missing. In future studies, digital sport offers also need to be examined regarding the risk of injury. We are unable to comment based on our data. However, considering the benefits and limitations of digital sport offers, it’s important to be noted that especially in CrossFit®, training at home without an on-site trainer may lead to increased injury rates [38].

## Conclusions

The reason for our study was the closure of fitness facilities purposing social distancing during the first lockdown, which appears to be an essential step to slow down the spread of SARS-CoV-2. However, it is still unclear what role fitness facilities and CrossFit® training centers play in terms of distribution. Moreover, the authors Gil et. all demonstrate that physical strength and increased muscle power, which can be improved by both CrossFit® and weightlifting, allows a better recovery from a COVID-19 infection [39]. Therefore, we emphasize the importance of maintaining exercise and training behavior, e.g., through digital sports offers as shown by our data, especially in times of global pandemic.

Our study shows that the changes in the training behavior of CFA and WLA due to the restrictions to combat the spread of SARS-CoV-2 have opened opportunities for CrossFit® and weightlifting sports which may become even more important in the future. So, it opened new opportunities for training facilities to expand their offerings and reach more potential customers through digital sports services. Our data show that especially among CFA, digital sports offerings were accepted across all age groups. Since digital training can be participated from anywhere, we assume the importance will increase in the future due to business trips, vacations, increasing mobility. In summary, digital sports offers every athlete the opportunity to practice CrossFit® and benefit from positive effects on health, regardless of their location and regardless of whether there is a training center on location.

## Data Availability

The datasets used and analyzed during the current study are available from the corresponding author on reasonable request.

## Abbreviations

CFA: CrossFit® athletes
CF5: CrossFit® athletes who lost 5 kg body mass
HIIT: High-intensity interval training
KMO: Kaiser–Meyer–Olkin
SD: Standard devison
WLA: Weightlifting athletes
WOD: Workout of the day
